# Superficial Bound of the Depth Limit of Two-Photon Imaging in Mouse Brain

**DOI:** 10.1523/ENEURO.0255-19.2019

**Published:** 2020-01-16

**Authors:** Kevin Takasaki, Reza Abbasi-Asl, Jack Waters

**Affiliations:** Allen Institute for Brain Science, Seattle, WA 98109

**Keywords:** cortex, 2-photon, 3-photon, multiphoton, fluorescence

## Abstract

Two-photon fluorescence microscopy has been used extensively to probe the structure and functions of cells in living biological tissue. Two-photon excitation generates fluorescence from the focal plane, but also from outside the focal plane, with out-of-focus fluorescence increasing as the focus is pushed deeper into tissue. It has been postulated that the two-photon depth limit, beyond which results become inaccurate, is where in-focus and out-of-focus fluorescence are equal, which we term the balance depth. Calculations suggest that the balance depth should be at ∼600 µm in mouse cortex. Neither the two-photon depth limit nor the balance depth have been measured in brain tissue. We found the depth limit and balance depth of two-photon excitation in mice with GCaMP6 indicator expression in all layers of visual cortex, by comparing near-simultaneous two-photon and three-photon excitation. Two-photon and three-photon results from superficial locations were almost identical. two-photon results were inaccurate beyond the balance depth, consistent with the depth limit matching the balance depth for two-photon excitation. However, the two-photon depth limit and balance depth were at 450 µm, shallower than predicted by calculations. Our results were from tissue with a largely homogenous distribution of fluorophores. The expected balance depth is deeper in tissue with fewer fluorophores outside the focal plane and our results therefore establish a superficial bound on the two-photon depth limit in mouse visual cortex.

## Significance Statement

This study measures the maximum depth in the mouse brain to which it is possible to obtain quantitatively accurate results with two-photon microscopy, a form of non-linear fluorescence microscopy.

## Introduction

Two-photon excitation permits fluorescence imaging with cellular and subcellular resolution hundreds of micrometers into biological tissue. Generally, the maximal imaging depth (depth limit) of two-photon excitation is determined by fluorescence from outside the focal plane. As the focal plane is pushed deeper into scattering tissue, illumination intensity at the tissue surface must be increased to maintain intensity in the focal plane, resulting in an increase in out-of-focus fluorescence with increasing depth (Ying et al., 1999; Theer et al., 2003). In a seminal study, Theer and Denk (2006) explored two-photon excitation analytically, calculating the expected in-focus and out-of-focus fluorescence under different conditions, including at different depths. Theer and Denk (2006) arbitrarily identified the two-photon depth limit as the depth at which the detected fluorescence generated by ballistic and scattered excitation light outside the focal plane equals that from fluorophores excited in the ballistic focus. The ratio of in-focus and out-of-focus fluorescence is a complex function of numerous factors, including numerical aperture, laser pulse duration, scattering anisotropy, and fluorophore distribution, but the calculations of Theer and Denk (2006) suggest that in-focus and out-of-focus fluorescence are equal at approximately three scattering length constants under typical imaging conditions. In rodent cortical gray matter, three scattering length constants corresponds to ∼600 µm below the tissue surface.

Three-photon excitation permits deeper imaging than two-photon excitation, in part because three-photon excitation generates fluorescence almost exclusively from the focal plane (Kobat et al., 2009, 2011; Horton et al., 2013; Ouzounov et al., 2017; Yildirim et al., 2019). In the absence of out-of-focus fluorescence, one expects the functional properties of neurons measured with two-photon and three-photon excitation to be identical, but the impact of out-of-focus fluorescence has not been measured. three-photon excitation offers the opportunity to estimate in-focus and out-of-focus fluorescence and thereby test the predictions of earlier analyses. We implemented near-simultaneous two-photon and three-photon excitation to compare results 200–650 µm below the surface of the brain in transgenic mice with dense GCaMP6 expression throughout neocortex. Our results indicate that two-photon and three-photon excitation produce equivalent results in superficial layers but not in deep in cortex, that the depth limit of two-photon excitation is where in-focus and out-of-focus fluorescence are equal, and that this depth is 450 µm.

## Materials and Methods

### Basic three-photon microscope

Our three-photon microscope was built around a Coherent Monaco/Opera-F laser source (≤2 nJ, 50 fs pulses at 1 MHz; Coherent Inc.) and a modified MIMMS microscope manufactured by Sutter Instrument. We replaced the scan and tube lenses (respectively, Thorlabs SL50-3P and a Plössl pair of achromatic doublets, Thorlabs AC254-400-C) to improve transmission at 1300 nm. The primary dichroic mirror was FF735-DI02 (Semrock). We used an Olympus 25×/1.05 objective (75% transmission at 1300 nm) or Nikon 16×/0.8 objective (50% transmission at 1300 nm) and image acquisition was controlled by ScanImage (Vidrio Technologies LLC) with acquisition gating for low rep rate lasers.

We estimated group delay dispersion (GDD) through the microscope at ∼15,000 fs^2^, approximately half of which was attributable to the Pockels cell (360-40-03-LTA, Conoptics Inc). To compensate, we built a four-pass pulse compressor using a single SF-11 glass prism (Thorlabs PS-853) and a two hollow roof prism mirrors (Thorlabs HRS1015-P01 and HR1015-P01). Compression was tuned by maximizing brightness with a fluorescein sample.

Here, 400–500 mW of 1300 nm illumination was available after the objective, corresponding to transmission from laser source to sample of ∼20%. The maximum field of view of three-photon excitation was 360 × 360 µm. Images were acquired with dual linear galvanometers at a frame rate of ∼8 Hz.

### Illumination intensity

Photodamage is often a concern in light microscopy. Photodamage can result from linear processes, principally heating (resulting from the absorption of infrared light by water in brain tissue) and from non-linear processes. Non-linear processes are of particular concern with high-energy pulsed sources such as those used for two-photon and three-photon fluorescence microscopy. Heating-related photodamage often occurs with >250 mW of prolonged illumination at 800–1040 nm (Podgorski and Ranganathan, 2016) and the molar extinction coefficient of water at 1300 nm is ∼2× that at 900 nm (Curcio and Petty, 1951; Hale and Querry, 1973; Bertie and Lan, 1996), suggesting that heating-related tissue damage may occur at more than ∼100–150 mW of prolonged illumination at 1300 nm. To avoid damage, we used illumination intensities <100 mW. Typically, we could image through the depth of neocortex using <30-mW illumination while maintaining a signal-to-noise ratio comparable to typical two-photon experiments. We rarely observed signs of photodamage, even in mice subjected to 2 h of continuous three-photon imaging per day for 5 d.

### Near-simultaneous two-photon and three-photon excitation

For two-photon excitation, we used a Coherent Chameleon Ultra II laser source at 920 nm. For near-simultaneous two-photon and three-photon excitation, we used a Nikon 16×/0.8 objective (50% transmission at 1300 nm). Time-averaged power available after the objective was 200–250 mW at 1300 nm. To match the focal planes of two-photon and three-photon excitation, to the two-photon path, we added an electrically-tunable lens (EL-10-30-TC, Optotune).

### Mice and surgeries

We used Cre-lox transgenic mice to drive GCaMP6s expression in excitatory neurons throughout cortical layers and areas, crossing Emx1-IRES-Cre (B6.129S2-*Emx1tm1(cre)Krj*/J, JAX stock number 005628; Gorski et al., 2002) or Slc17a7-IRS2-Cre (B6;129S-*Slc17a7^tm1.1(cre)Hze^*/J, JAX stock number 023527; Harris et al., 2014) and Ai162(TIT2L‐GCaMP6s‐ICL‐tTA2 reporter mice (JAX stock number 031562; Daigle et al., 2018).

A chronic cranial window was implanted over visual cortex as described previously (Goldey et al., 2014; de Vries et al., 2018). Briefly, under 0.5–2% isoflurane anesthesia, a head restraint bar was attached to the skull using C & B Metabond (Parkell) and a 5-mm diameter craniotomy was opened over the left visual cortex at coordinates 2.7 mm lateral, 1.3 mm anterior to lambda. A durotomy was performed and the craniotomy was sealed with a stack of three #1 coverslips, attached to each other using optical adhesive, and attached to the skull with Metabond.

### Visual stimuli

Visual stimuli were full-field sinusoidal gratings of six orientations, each drifting perpendicular to its orientation (12 directions), at spatial frequencies of 0.04 and 0.08 cycles per degree and a temporal frequency of 1 Hz. Each grating was presented 8 times in random order, each for 2 s with 1 s of gray screen between presentations; 0° corresponds to a grating drifting horizontally in the nasal-to-temporal direction and 90° to a downward-drifting grating. The visual stimulus display and its calibration were as described previously (de Vries et al., 2018). Briefly, stimuli were displayed on an LCD monitor, 15 cm from the right eye, gamma-corrected, and of mean luminance of 50 cd/m^2^. Spherical warping was employed to ensure the apparent size, speed, and spatial frequency were constant across the monitor.

### Image analysis

Image analysis was performed using custom routines in Python 3. For comparison of two-photon and three-photon excitation, images were first separated into two-photon and three-photon movies. Dark current, the mean of several images acquired with no laser illumination, was measured in each movie and subtracted. Image brightness was measured in digitizer units. To avoid artifacts, each movie was normalized to the same mean brightness.

Image contrast was expressed on a scale from 0 (no contrast) to 1. Contrast was calculated locally (in 22.5 × 22.5-pixel blocks) from the temporal mean projection of a movie, the final value being the mean of all the blocks. Contrast in each block was defined as 1, minimum brightness/maximum brightness.

Each movie was motion-corrected and putative neuronal somata identified by segmentation. Soma and neuropil fluorescence traces were extracted and neuropil fluorescence was subtracted from the corresponding soma trace (r = 1). Motion correction, segmentation and trace extraction were performed using Suite2p (Pachitariu et al., 2017) with default settings except for maxregshift which was set to 0.2 to permit less than or equal to ∼70-µm motion correction in each transverse axis. We manually checked trace extraction for a small sample of neurons by applying spatial masks to motion-corrected fluorescence movies. Neuropil subtraction, calculation of ΔF/F, averaging of traces and identification of direction preference were performed in Python. The preferred direction for each neuron was defined as the direction that evoked the largest mean peak change in ΔF/F.

Motion correction was the mean of *x*- and *y*-corrections applied by Suite2p. Neuron count was the number of putative somata returned by Suite2p, with manual editing to assist the sorting of somatic from non-somatic regions of interest; % match was the percentage of putative neurons segmented in the three-photon image that were also segmented in the corresponding two-photon image, assessed manually by comparing images of segmented regions. Pearson correlation coefficient was calculated from the neuropil-subtracted fluorescence traces using scipy.stats.pearsonr. To ensure that the correlation coefficient calculation was from matching regions of interest, traces were extracted from two-photon and three-photon movies using regions of interest segmented from three-photon movies.

To compare two-photon and three-photon measurements of responses to drifting gratings, we used two measures: mean fluorescence response and preferred direction. Again, these measures were applied to traces extracted from two-photon and three-photon movies using regions of interest segmented from three-photon movies.

### Experimental design and statistical analysis

Results supporting Figure 3 were derived from two mice, one male and one female. A total of 1145 regions of interest, corresponding to putative somata, were identified in images from these two mice.

### Modeling in-focus and out-of-focus fluorescence

To estimate the out-of-focus fluorescence generated by excitation light focusing through a homogeneous volume of fluorescent and scattering tissue, we modeled the intensity of ballistic and scattered light, $I_{b}\left(z,\rho \right)$ and $I_{s}\left(z,\rho \right)$, respectively, in a plane transverse to the optical axis defined by the polar radius, ρ, and depth z below the surface of the brain. We calculated the out-of-focus, two-photon-excited fluorescence (F_oof_) numerically, following Theer and Denk (2006),

$$F_{oof}=C_{2p}\int_{V}{\left[I_{s}\left(z,\rho \right)+I_{b}\left(z,\rho \right)\right]}^{2}dV,$$

where V is the out-of-focus illuminated volume of tissue,$C_{2p}$ is a modality-specific scaling factor incorporating contributions from fluorophore concentration and excitation efficiency, and assumed to be constant over the volume.

We neglected possible depth dependence of fluorescence collection and detection, non-conservative attenuation due to bulk absorption of near-IR light, and the time dependence of excitation by ultrashort pulses that becomes a significant factor for pulse widths less than ∼50 fs (Theer and Denk, 2006; but see also Leray et al., 2007).

Previous models (Xu and Webb, 1996; Theer and Denk, 2006) neglected the difference in distances traveled through tissue by on-axis and marginal rays. The difference in distance can be substantial for high-numerical aperture objectives, but of marginal importance when the focal plane is many multiples of the scattering length below the tissue surface. Here, we calculated fluorescence with the focal plane one to four scattering lengths below the tissue surface and therefore account for the dependence on propagation angle relative to the optical axis by incorporating a radially varying propagation distance,

$$s\left(z,\rho \right)=z\sqrt{1+\frac{\rho^{2}}{{\left(z_{0}-z\right)}^{2}}},$$

where $z_{0}$ is the focal plane depth. This factor modifies the intensity profiles of $I_{b}$ and of $I_{s}$.$F_{oof}$ can be decomposed into individual contributions from ballistic, scattered, and cross-term interaction excitation, for two-photon excitation:

(1) $$F_{oof}=\int_{Voof}^{}dz\int_{A}\left[I_{b}^{2}\left(z,\rho \right)+I_{s}^{2}\left(z,\rho \right)+2I_{s}\left(z,\rho \right)I_{b}\left(z,\rho \right)\right]dA=\int_{Voof}^{}\left[F_{b}\left(z\right)+F_{s}\left(z\right)+F_{sb}\left(z\right)\right]dz,$$

where, $V_{oof}$ is the out-of-focus volume denoting the range $\left(-\infty ,z_{0}-\delta \right)\cup \left(z_{0}+\delta ,\infty \right)$, δ is the exclusion depth of in-focus light around $z_{0}$.

In our calculations, δ was a fifth of the scattering length, or 40 μm, which we assume to be larger than the depth of focus and therefore underestimates the magnitude of the background; wavelength was 900 nm; numerical aperture 0.8; and anisotropy factor 0.9.

To calculate ballistic and scattered light intensities, we considered a Gaussian beam propagating from the surface (z=0) of a scattering medium of scattering length l=1a to a ballistic focus located at $z=z_{0}$. We introduced a direction dependent propagation length $s\left(z,\rho \right)=z\sqrt{1+\frac{\rho^{2}}{{\left(z_{0}-z\right)}^{2}}}$, and calculate the ballistic intensity profile at depth z and radial distance ρ according to

$$I_{b}\left(z,\rho \right)=\frac{2P_{0}}{\pi w^{2}\left(z\right)}\exp\left[\frac{-2\rho^{2}}{w^{2}\left(z\right)}\right]\exp\left[-as\left(z,\rho \right)\right],$$

where, $w\left(z\right)=2\sqrt{\frac{\lambda \left({\left(z_{0}-z\right)}^{2}+z_{R}^{2}\right)}{4\pi nz_{R}}}$ is the 1e2 width, $z_{R}=\frac{\lambda }{n\pi \tan^{2}\theta }$ is the Rayleigh length determined by the NA-derived focusing half-angle.

As in Theer and Denk (2006), we calculated the intensity distribution of scattered light from a beam spread function derived for small-angle scattering (McLean et al., 1998). We integrated over temporal and angular coordinates to obtain the normalized spatial distribution function

$$h\left(z,\rho \right)=\frac{3n}{\pi az^{3}〈\Theta^{2}〉}\exp\left[-\frac{3n\rho^{2}}{az^{3}〈\Theta^{2}〉}\right].$$

The spreading parameter $〈\Theta^{2}〉=2\left(1-g\right)$ is derived from the anisotropy factor g and the function h(z,ρ) accounts for the diffusive spreading of scattered light with increasing depth from an initial on-axis ray, with total power increasing with depth according to 1−exp⁡[−az], modeling the transfer of energy from the ballistic to the scattered field.

Integrating over the initial surface distribution, the intensity distribution of scattered light at depth z is

$$I_{s}\left(z,\rho \right)=\overset{2\pi }{\underset{0}{\int }}d\varphi \overset{\infty }{\underset{0}{\int }}\frac{2P_{0}\beta }{\pi^{2}w_{0}^{2}}exp\left[-\frac{2\eta^{2}}{w_{0}^{2}}\right]\exp\left[-\beta \left(\rho^{2}+{\left(\frac{z_{0}-z}{z_{0}}\right)}^{2}\eta^{2}-2\rho \left(\frac{z_{0}-z}{z_{0}}\right)\eta \cos\varphi \right)\right]\left(1-\exp\left[-as_{0}\right]\right)\eta d\eta ,$$

where, $\beta \equiv \frac{3n}{as_{0}^{3}〈\Theta^{2}〉}$, $s_{0}=z\sqrt{1+\frac{\eta^{2}}{z_{0}^{2}}}$ is the propagation distance from the surface, and $w_{0}=2\sqrt{\frac{\lambda \left(z_{0}^{2}+z_{R}^{2}\right)}{4\pi nz_{R}}}$ is the Gaussian beam width at the surface.

### Proportion of fluorescence originating from the focal plane

Calculation of the ratio of in-focus and out-of-focus fluorescence was based on image contrast. We subdivided the 256 × 512-pixel images of the motion-corrected, time-averaged two-photon and three-photon movies into 32 × 32-pixel subregions. Within each subregion, we determined the minimum pixel value and pixel value mean, $min_{n}\left(\bar{F}\right)$ and ${〈\bar{F}〉}_{n}$ , respectively, where $\bar{F}$ denotes the time-averaged fluorescence in each pixel with the minimum and mean functions over the 32 × 32 = 1024 pixels. For each subregion in each imaging modality (two-photon and three-photon), we then calculated a contrast parameter, $\gamma_{j,k}\equiv \frac{〈\bar{F}〉-\min\left(\bar{F}\right)}{〈\bar{F}〉}$, for the j-th subregion in the k= {2,3} (two-photon, three-photon) modality.

To calculate in-focus and out-of-focus fluorescence, we made three assumptions. First, we assumed the time-averaged fluorescence in each pixel reflects the sum of the in-focus and out-of-focus fluorescence ($\bar{F}=\bar{F_{i}}+\bar{F_{oof}}$). Second, we assumed three-photon excitation generates no out-of-focus fluorescence so that $\bar{F}=\bar{F_{i}}$ for three-photon excitation. Third, we assumed in-focus fluorescence is proportional to a modality-independent concentration factor, C, with a modality-dependent proportionality constant, so that ${F_{i}}_{k}=\alpha_{k}C$.

Hence $\gamma_{j,3\text{p}}=\frac{〈\bar{C}〉-\min\left(\bar{C}\right)}{〈\bar{C}〉}$ and $\gamma_{j,\text{2p}}=\frac{\alpha_{\text{2p}}\left(〈\bar{C}〉-\min\left(\bar{C}\right)\right)}{\alpha_{\text{2p}}〈\bar{C}〉+F_{oof}}$.

As a measure of the percentage of fluorescence that originates from the focal plane, we calculated an empirical contrast ratio (ECR): $\text{ECR}\equiv \frac{\gamma_{j,\text{2p}}}{\gamma_{j,\text{3p}}}=\frac{\alpha_{\text{2p}}〈\bar{C}〉}{\alpha_{\text{2p}}〈\bar{C}〉+F_{oof}}=\frac{\bar{F_{i}}}{\bar{F_{i}}+\bar{F_{oof}}}$.

The ECR calculated in each subregion was averaged over the subregions to determine the time-averaged ECR for a given imaging depth.

We calculated the theoretical contrast ratio via a signal-to-background ratio calculation. We modeled the total in-focus fluorescence, $F_{i}$, according to $F_{i}=\frac{{〈P_{z0}〉}^{2}\pi }{\lambda }$ where $P_{z0}$ is the total, scattering attenuated, ballistic power estimated at the focal plane according to $P_{z0}=2\pi \int_{0}^{\infty }I_{b}\left(z_{0},\rho \right)\rho d\rho$ . The signal-to-background ratio was defined as the ratio of total in-focus to out-of-focus fluorescence, given by $SBR\equiv \frac{F_{i}}{F_{oof}}$ which ranges from 0 at very large depths to ∞ in the background-free case. We defined the contrast ratio, $CR\equiv \frac{F_{i}}{F_{i}+F_{oof}}=\frac{SBR}{1+SBR}$ to range from 0 to 1.

## Results

**Figure 1.**
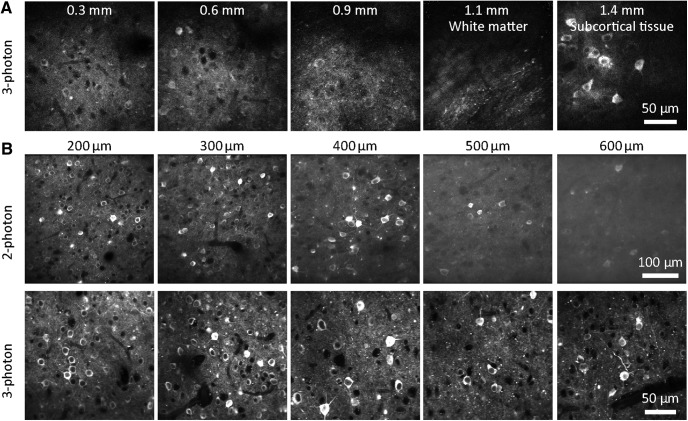
Contrast declines with depth with two-photon excitation. ***A***, Example three-photon images from 300, 600, 900, 1100, and 1400 µm below the pial surface of visual cortex. Emx1-IRES-Cre;CaMK2a-tTA;Ai94 mouse. ***B***, Comparison of images acquired from a single Emx1-IRES-Cre;CaMK2a-tTA;Ai94 mouse (different fields of view) using two-photon and three-photon excitation, focused 200–600 µm below the pial surface of visual cortex.

As an illumination source for three-photon excitation, we used a 40-W Coherent Monaco laser source and Opera-F optical parametric amplifier, providing 2 µJ, 50-fs pulses at 1 MHz. We configured a MIMMS two-photon microscope for three-photon excitation, exchanging the scan and tube lens to increase transmission through the microscope at 1300 nm and added a compressor to compensate for pulse dispersion between the laser source and sample. Through a cranial window over visual cortex, we were routinely able to image neurons >1 mm below the pial surface of cortex in GCaMP6 mice (Fig. 1*A*). Fluorescence intensity followed a cubic relationship with illumination intensity, consistent with fluorescence being driven by the absorption of three photons.

**Figure 2.**
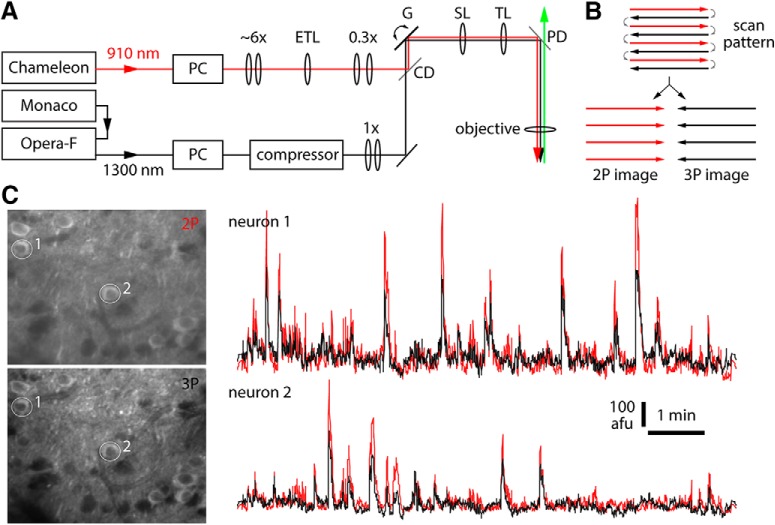
Implementation of near-simultaneous two-photon and three-photon excitation. ***A***, Schematic of the optical layout for near-simultaneous two-photon and three-photon excitation; 1300-nm beam (black) passed a Pockels cell (PC), prism compressor, a collimating telescope, combining dichroic mirror (CD), x-y galvanometer pair (G), scan lens (SL), tube lens (TL), FF735-DI02 primary dichroic mirror (PD), and objective lens; 910-nm beam (red) passed a PC, beam expansion to ∼1 cm in diameter, electrically-tunable lens (ETL), 0.3× beam expansion before being reflected by the combining dichroic mirror onto the galvanometer pair. ***B***, Scanning strategy for near-simultaneous two-photon and three-photon excitation. Red: 920-nm excitation, no 1300-nm excitation. Black: no 920-nm excitation, 1300-nm excitation. Gray: both lasers blocked. Lines were sorted into two-photon and three-photon images. ***C***, Example images from 350 µm below the pia, with traces from two somata.

In mice expressing GCaMP broadly in cortical pyramidal neurons, loss of contrast was noticeable in two-photon images from hundreds of micrometers below the brain surface, where contrast was preserved by three-photon excitation (Fig. 1*B*). To compare two-photon and three-photon excitation more directly, we implemented near-simultaneous two-photon and three-photon excitation. We used two laser sources, combining the beams immediately before the scanning galvanometers (Fig. 2*A*). With a fast Pockels cell on each laser line acting as a shutter, we alternated two-photon and three-photon excitation, line-by-line (Fig. 2*B*). The line duration was 0.5 ms, resulting in 0.5 ms separation of two-photon and three-photon images.

**Figure 3.**
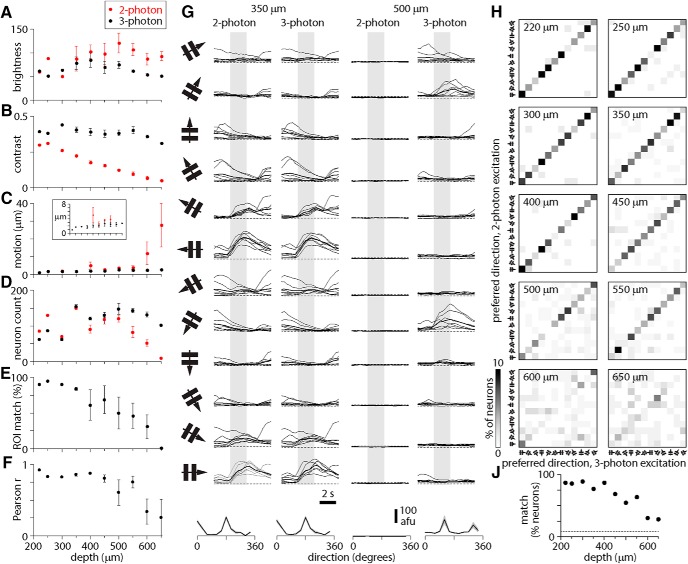
Changes in two-photon image quality and apparent ΔF with depth. ***A–D***, Plots of image brightness (***A***), contrast (***B***), corrected motion (***C***), and ROI count (***D***) for two-photon (red) and three-photon excitation (black), plot as a function of depth below the pial surface of cortex. Mean ± SEM of three experiments from two Slc17a7-Cre;Ai162 mice. ***E***, ROI match (percentage of three-photon ROIs also segmented from two-photon images) as a function of depth. ***F***, Pearson correlation coefficient between two-photon and three-photon fluorescence traces, plot as a function of depth. ***G***, Two-photon and three-photon changes in fluorescence to grating stimuli for two neurons, 350 and 500 µm below the pia. Each panel shows change in fluorescence (in arbitrary fluorescence units) through time during presentation of the drifting grating (icon to left indicates orientation and direction) for 2 s (gray bar). Eight individual traces and the mean (thick line) per direction. Dashed line indicates zero fluorescence. Below, Resulting direction tuning curve. ***H***, Plots comparing preferred direction of neurons measured with two-photon (*y*-axis) and three-photon (*x*-axis) excitation, for each depth. Colors indicate percentages of the total number of neurons at each depth (zero is white, 10% is black, see color bar). Directions progress at 30° intervals from the low left corner of each plot (icons). ***J***, Percentage of neurons with matching direction preferences measured with two-photon and three-photon excitation, from 200 to 650 µm. Dashed line: 8.3%.

In superficial cortex, two-photon and three-photon results were similar. The same neurons were visible in near-simultaneous two-photon and three-photon images and changes in fluorescence were coincident in two-photon and three-photon image pairs (Fig. 2*C*; Movie 1); the results of motion correction and segmentation on two-photon and three-photon movies were similar (SDs of motion correction distributions <2 µm at <350 µm; Fig. 3*C*); there were 50–90 neurons identified in each image (Fig. 3*D*); >80% of neurons in three-photon images matched a neuron in the corresponding two-photon image (Fig. 3*E*); and traces extracted from matching neurons in two-photon and three-photon movies were strongly correlated, with Pearson correlation coefficients of ∼0.8–0.9 (Fig. 3*F*), consistent with previous studies (Wang et al., 2017; Ouzounov et al., 2017, 2019).

Movie 1. Examples of simultaneous two-photon and three-photon image pairs at different depths. Examples of matched two-photon and three-photon movies 250, movies 450, and movies 650 μm below the pia. Two-photon and three-photon movie pairs were acquired pseudo-simultaneously. Each movie was acquired at a different illumination intensity and each was scaled differently for display purposes. Slc17a7-Cre;Ai162 mouse. Movies acquired at 8 Hz. Playback at 20 Hz. 10.1523/ENEURO.0255-19.2019.video.1

The similarity of two-photon and three-photon results declined with depth. In three-photon images, image contrast, motion correction, and number of neurons changed little with depth. In two-photon images, contrast declined incrementally with depth, to near zero at 650 µm (Fig. 3*B*). Lateral motion correction from two-photon movies increased with depth: the SD of motion correction was <3 µm at <400 µm; at 650 µm, the SD of lateral motion correction was <3 µm for three-photon excitation and ∼25 µm for two-photon excitation (Fig. 3*C*). The segmentation routine identified few neurons in deep locations (Fig. 3*D*), and the overlap between matching neurons in two-photon and three-photon images and the correlation coefficient between the resulting traces both declined at >350–400 µm (Fig. 3*E*,*F*).

To determine how the decline in image quality with depth affects the functional properties of cortical neurons measured with two-photon excitation, we examined the apparent responses of cortical neurons to visual stimuli. We presented sinusoidal gratings drifting in 12 directions and calculated the direction preference of each neuron from extracted fluorescence traces, comparing results from two-photon and three-photon excitation. For superficial neurons, visually-evoked changes in two-photon and three-photon fluorescence were almost identical, trial-by-trial (Fig. 3*G*) and the resulting preferred direction of each neuron was closely matched (Fig. 3*H*), with 83% (305 of 368) of neurons ≤350 µm from the brain surface exhibiting identical preferred directions with two-photon and three-photon excitation. The percentage of neurons with matching two-photon and three-photon direction preference declined with depth and at 600 µm, the number of neurons with matching preference was above chance (1/12 = 8.3%), but ≪50%.

**Figure 4.**
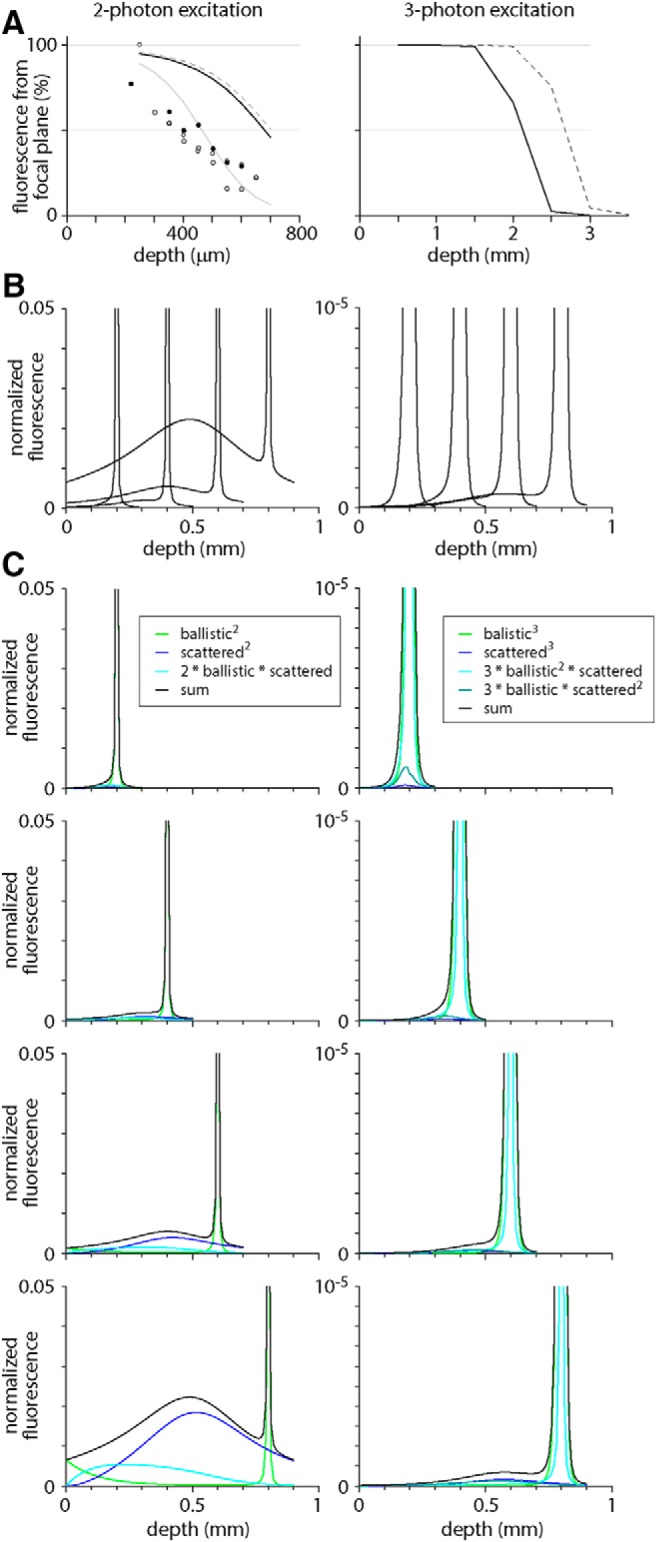
In-focus and out-of-focus fluorescence. ***A***, Percentage of total fluorescence that originates from the focal plane, plot as a function of depth of the focal plane below the brain surface. Each point represents a single measurement (from a movie at one depth in one mouse). Lines are calculated from Equation 1 with scattering length constants of 200 µm (black) and 150 µm (gray). Dashed lines are relationships from the literature for scattering lengths constants of 200 µm: Equation 4 of Theer and Denk (2006) for two-photon excitation and Equation 7 of Xu and Webb (1996) for ballistic three-photon excitation. ***B***, Plots showing the depth from which fluorescence originates with the focal plane at 200, 400, 600, and 800 µm below the brain surface. Fluorescence was calculated with Equation 1 and normalized to that in the focal plane. Note the difference in scale for two-photon and three-photon excitation. ***C***, Breakdown of sources underlying the total fluorescence in panel ***B*** (Theer and Denk, 2006). Two-photon: 900-nm illumination, scattering length 200 µm (Eq. 1). Three-photon: 1300-nm illumination, scattering length 200 µm, equivalent formulation. Colors: fluorescence from ballistic incident photons (light green), from scattered photons (dark blue) and from a mixture of ballistic and scattered photons (cyan and deep green). Black: the sum of all fluorescence sources (reproduced in panel ***B***).

Two-photon and three-photon excitation produce equivalent results from superficial depths, but the results become less similar >400 µm below the brain surface. Increasing out-of-focus fluorescence and the resulting decline in image contrast are the likely cause. From the ratio of contrast in two-photon and three-photon images, we estimated the percentage of fluorescence that originated from the focal plane during two-photon excitation. As expected, the percentage of two-photon fluorescence originating from the focal plane decreased with increasing depth (Fig. 4*A*). In-focus and out-of-focus fluorescence were equal at ∼400–450 µm, the depth beyond which the results of two-photon excitation are inaccurate. Hence our results support the depth limit corresponding to the depth at which in-focus and out-of-focus fluorescence are equal.

We compared our measurements of in-focus and out-of-focus fluorescence with predictions from theoretical modeling of focused light propagation in scattering tissue (Theer and Denk, 2006). According to this model, 50% in-focus fluorescence occurs approximately three scattering lengths below the brain surface, at 600–700 µm for a scattering length of 200 µm (Fig. 4*A*). Our measurements indicate that in-focus and out-of-focus fluorescence are equal at ∼450 µm. Three-photon excitation is almost free of out-of-focus fluorescence at these depths.

We expect two-photon excitation to support imaging >450 µm below the brain surface if there are few fluorophore molecules outside the focal plane. Unfortunately, out-of-focus fluorescence arises from fluorophores throughout the tissue above and, to a lesser extent, below the focal plane (Fig. 4*B*; Theer and Denk, 2006). A reduction in the number of molecules near the brain surface would likely have limited impact on out-of-focus fluorescence and the two-photon depth limit.

## Discussion

We compared the results of two-photon and three-photon excitation of GCaMP6s in excitatory neurons in mouse visual cortex. As expected (Ouzounov et al., 2019), results from superficial cortex were similar, suggesting that neither two-photon nor three-photon images were compromised by saturation or phototoxic effects. With increasing depth from ∼250 to 650 µm, two-photon image contrast declined and three-photon image contrast was preserved. Many measures (estimated motion, number of neurons segmented, matching of segmented neurons, correlation traces, similarity of fluorescence changes, similarity of preferred direction) were robust to changes in two-photon image contrast to ∼400 µm, but deteriorated between 400 and 550 µm on average, some abruptly, compromising measurement of fluorescence changes and direction tuning.

In our experiments, we used a mouse line with GCaMP6s expression in excitatory neurons through all layers of cortex. From the perspective of out-of-focus fluorescence, we expect these mice to be a worst-case scenario for two-photon excitation. In these mice, our results place the depth limit at ∼450 µm below the brain surface, shallower than the depth predicted by Theer and Denk (2006) and by our calculations. There are several factors that likely contribute to the mismatch of calculations and measurements. Aberrations are present in any imaging system, but not included in our or calculations or those of Theer and Denk (2006). We expect aberrations to reduce the depth at which in-focus and out-of-focus fluorescence are equal. Slight compression of cortex is common in cranial window preparations (de Vries et al., 2018) and might further reduce the depth limit by reducing the scattering length of cortical tissue. Hence one expects the measured depth limit of two-photon excitation to be shallower and our measurements indicate that the depth limit can be as shallow as ∼450 µm.

Our results drive two predictions that we have not tested directly. First, we expect that two-photon excitation will be adequate for characterization of functional properties such as direction tuning in neurons ≤450 µm from the brain surface in nearly all GCaMP6s mouse lines. Second, we expect two-photon and three-photon results to be comparable at >450 µm in many preparations. We observed substantial mouse-to-mouse variability at 500–650 µm, suggesting that two-photon excitation might be a viable tool to >450 µm in a small subset of our mice. In other mouse lines and tissues, two-photon excitation at >450 µm will provide more accurate functional measurements in preparations with less out-of-focus fluorescence, including tissues with sparser expression of GCaMP6s and tissues labeled with indicators with low resting fluorescence, such as jGCaMP7c (Dana et al., 2018). In such tissues, out-of-focus fluorescence will be reduced, but will still occur and may equal in-focus fluorescence at a location deeper than 450 µm. With sufficiently sparse labeling, out-of-focus fluorescence may be insignificant at all depths, in which case the depth limit will likely be set by the thermal limit of brain tissue (Podgorski and Ranganathan, 2016).

There are several strategies that can extend the depth limit of two-photon microscopy. Neuronal structure and activity can be imaged at greater depth with red-shifted fluorophores (Kobat et al., 2009; Tischbirek et al., 2015; Kondo et al., 2017). The increased depth results from reduced scattering of illuminating photons (Wang et al., 2019), which increases intensity in the focal plane and reduces the interaction of scattered photons, the primary source of out-of-focus fluorescence. Aberration correction, such as with adaptive optics, can increase the depth limit. Aberrations are almost inevitable when imaging into intact brain and expand the point spread function (Ji et al., 2010, 2012; Liu et al., 2019), reducing in-focus but not out-of-focus fluorescence. One might expect aberration correction to extend the depth limit toward, but not beyond three scattering length constants, the depth at which in-focus and out-of-focus fluorescence are predicted to be equal in calculations that take no account of aberrations (Theer and Denk, 2006). Finally, reducing the pulse duration to <20 fs is expected to increase the in-focus to out-of-focus fluorescence ratio, increasing the depth limit (Theer and Denk, 2006), and where peak pulse energy or heating are limiting factors, more modest changes in pulse duration can increase imaging depth (Theer et al., 2003; Mittmann et al., 2011). Simply increasing illumination intensity, however, will increase in-focus and out-of-focus fluorescence equally, leaving the depth limit unaffected.

In summary, we have established that two-photon and three-photon excitation are equivalent less than or equal to ∼450 µm below the brain surface in mice with GCaMP6s expression throughout cortical layers. Tentatively, we suggest the depth limit of two-photon excitation is 450 µm or deeper in nearly all mouse lines, since few if any mice express a higher proportion of fluorophore molecules outside the focal plane than mice with expression throughout the cortical layers. In tissues with and tissues without extensive fluorophore expression outside the focal plane, three-photon excitation enables measurement of cellular activity beyond the depth limit of two-photon excitation.

## Synthesis

Reviewing Editor: Christian Hansel, University of Chicago Decisions are customarily a result of the Reviewing Editor and the peer reviewers coming together and discussing their recommendations until a consensus is reached. When revisions are invited, a fact-based synthesis statement explaining their decision and outlining what is needed to prepare a revision will be listed below. The following reviewer(s) agreed to reveal their identity: Jason MacLean. Note: If this manuscript was transferred from JNeurosci and a decision was made to accept the manuscript without peer review, a brief statement to this effect will instead be what is listed below.

The paper from the Allen Institute “Superficial bound of the depth limit of 2-photon imaging in mouse brain” submitted to eNeuro for publishing relies on the comparison of 3P and 2P excitation of fluorescent indicators in the mouse visual cortex for functional imaging. Using near simultaneous scanning of both 2P and 3P excitation the central claim is that the depth limit of 2P imaging is 450um below the cortical surface.

In general the idea of detailing the limits of a particular technique is helpful to a field, this manuscript suffers from a very limited view of the published work using either 2P or 3P excitation. The manuscript would be strengthened is the authors would place more caveats on their claims and acknowledge previous studies. The authors also rely heavily on the suite2p software for their analysis which as far as I can tell, never verified using other approaches. For example, it's not clear, to me at least, how the tuning of the neurons based the transients is established and then compared between the two excitation approaches.

Here are a few examples that would need some attention:

Line 315 “2-photon excitation will support imaging >450 µm below the brain surface if there are few fluorophore molecules outside the focal plane” ... Please cite the published studies that have shown it deep imaging using various modifications of 2P excitation. To name a few: (Mittmann et al., 2011, Tischbirek et al., 2015, Kondo et al., 2017). There are many methods for improving 2P imaging with the aim of imaging neuronal activity from deeper structures, at a minimum these need to be discussed, but ideally, experimentally explored.

Line 324: “confirms that the cellular signals reported by GCaMP6s are independent of the mechanism of excitation”. Not strictly correct as it strongly depends on the wavelength employed (Xu lab study from 2019).

In summary, it is well documented that 3P excitation enables deeper imaging of neuronal activity than 2P excitation, given all of the uncontrolled variables in the current study that certainly contribute to this outcome, it's not clear what this manuscript significantly adds to the field.
